# Injectable Composite Systems Based on Microparticles in Hydrogels for Bioactive Cargo Controlled Delivery

**DOI:** 10.3390/gels7030147

**Published:** 2021-09-18

**Authors:** Henrique Carrêlo, Paula I. P. Soares, João Paulo Borges, Maria Teresa Cidade

**Affiliations:** CENIMAT/i3N, Departamento de Ciência dos Materiais, NOVA School of Science and Technology, 2829-516 Caparica, Portugal; h.carrelo@campus.fct.unl.pt (H.C.); pi.soares@fct.unl.pt (P.I.P.S.); jpb@fct.unl.pt (J.P.B.)

**Keywords:** bioactive cargo, biomedical applications, drug delivery systems, hydrogels, microparticles

## Abstract

Engineering drug delivery systems (DDS) aim to release bioactive cargo to a specific site within the human body safely and efficiently. Hydrogels have been used as delivery matrices in different studies due to their biocompatibility, biodegradability, and versatility in biomedical purposes. Microparticles have also been used as drug delivery systems for similar reasons. The combination of microparticles and hydrogels in a composite system has been the topic of many research works. These composite systems can be injected in loco as DDS. The hydrogel will serve as a barrier to protect the particles and retard the release of any bioactive cargo within the particles. Additionally, these systems allow different release profiles, where different loads can be released sequentially, thus allowing a synergistic treatment. The reported advantages from several studies of these systems can be of great use in biomedicine for the development of more effective DDS. This review will focus on in situ injectable microparticles in hydrogel composite DDS for biomedical purposes, where a compilation of different studies will be analysed and reported herein.

## 1. Introduction

Drug delivery systems (DDS) can be defined as formulations that protect, transport, and release bioactive cargo, such as drugs or other agents (e.g., growth factors), to the human body. The release needs to be within a specific site and/or with a controllable and sustainable profile. Many types of bioactive agents have a fragile nature and can be rapidly cleared by the body. Their encapsulation within a DDS will protect them in the in vivo environment and prevent rapid clearance. DDS must also guarantee a sustainable release pattern that maintains therapeutic concentrations to the desired site. These systems have been developed for several purposes, particularly for applications in biomedicine [1].

There are different types of administration, such as oral, inhalation, or injection. Different routes can be used for an injection. This can be subcutaneous, intramuscular, intravenous, or intra-arterial [2]. Intravenous administration of pharmaceutical drugs has the downside of rapid clearance and general toxicity; for example, chemotherapeutic treatments are known to cause serious side effects. Many studies have developed DDS that can increase targetability and localized treatment. For example, different mechanisms are used to accumulate nanoparticles (either through active or passive targeting) in the tumour area [3]. To increase the treatment efficiency and reduce systemic toxicity, drugs must be released in situ.

In situ (or in loco) injection of DDS has also been the topic of different studies. Injection of a DDS to a specific site within the human body may offer some advantages. In loco, the system can directly release drugs/bioactive agents to the surrounding tissues without affecting healthy tissues, preventing unwanted cytotoxicity. This localised delivery will also be more effective because the drugs will be unloaded directly to the targeted site, acting more efficiently. This will avoid rapid clearance and the need for repeated administrations. Thus, toxic side effects can be diminished, or even avoided [4]. So, the design of DDS that can be injected and remain in loco is of great importance.

Hydrogels are 3D structures of interconnected chains of hydrophilic polymers, thus being great candidates for in situ injection. Water can penetrate through the hydrogel mesh pores and can be retained in large amounts. Hydrogels are also biocompatible and biodegradable. They also exhibit flexibility similarly to natural tissues, thanks to their water content, which is given by hydrophilic groups [5].

Hydrogel’s crosslinking can either be of physical or chemical nature. Physical cross-linking occurs with secondary forces, for example, hydrogen bonds between chains, hydrophobic interactions, or electrostatic ionic forces. These can be preceded by changes in pH, temperature, or ion presence. Physical crosslinking is reversible and non-toxic because it does not use any toxic chemical crosslinkers. However, mechanical properties are limited by weak bonds. Chemically crosslinked hydrogels have a matrix with covalently bonded chains, created, for example, by photo-crosslinking or Schiff’s base reactions. Chemical crosslinking results in strong bonds that are not reversible and may impact some toxicity to the hydrogel [6].

Hydrogels’ response to exterior stimuli is an important characteristic, especially in the so-called “smart hydrogels”. These react to external stimuli that will cause physical and/or chemical structural changes. For example, hydrogels can contract or swell with different pHs. In a drug loading scenario, these stimuli can be important to the cargo release. By controlling and adapting hydrogels’ characteristics, such as pore structure or crosslinking density, it is possible to control the unloading of bioactive agents from the hydrogel’s structure. Additionally, hydrogels can be injected in vivo and, due to their flexible nature, they will adjust their dimensions to the site. Thus, hydrogels are ideal materials to develop DDS [7].

**Figure 1:**
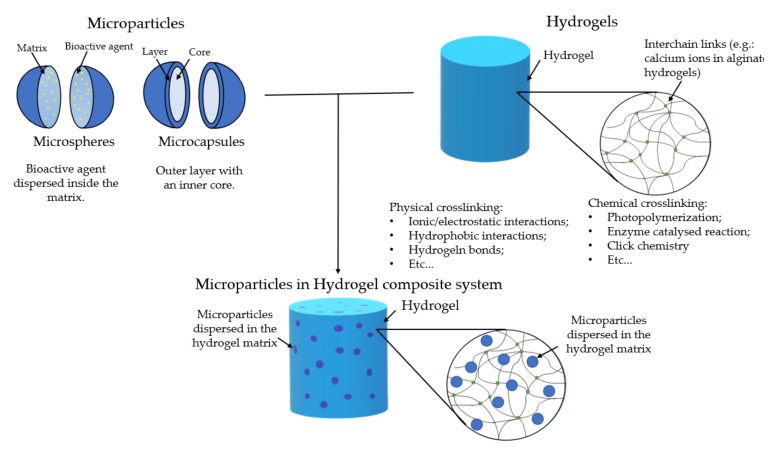
Schematic representation of microparticles (upper left), hydrogels (upper right), and the microparticles in hydrogel system (down).

Microparticles have also been used as DDS [8]. These can carry the cargo within their structure and can be tailored in a multitude of different ways. However, certain considerations must be accounted for; the difference between microsphere and microcapsule must be made (Figure 1). The former consists of a matrix where the drugs are dispersed homogenously. The latter is a shell layer that surrounds a reservoir core, which is where the cargo is held. In the literature, these two names are sometimes confused, and it is important to separate the two since they have different release profiles. Commonly, in microspheres, the permeation of water and the erosion of the polymeric matrix are important factors that will determine the release profile [9]. In microcapsules, the surface layer diffusion determines the release, and its rapid degradation might lead to a rapid release. There are other morphologies of microparticles, but these two are the most common [8].

The combination of microparticles and hydrogels as a composite system for bioactive cargo delivery has been the focus of different studies (Figure 1) [10,11]. This system will have advantages when compared to hydrogels or microparticles alone. The hydrogel will serve as a protective barrier and a carrier for drugs/bioactive agents and microparticles. Additionally, the hydrogel will serve as a double barrier for drugs/bioactive agents that are encapsulated within the particles. This will guarantee a more prolonged life of the system with a more sustainable release. Drug loading to the particles and/or to the hydrogel may allow a sequential release pattern.

**Figure 2:**
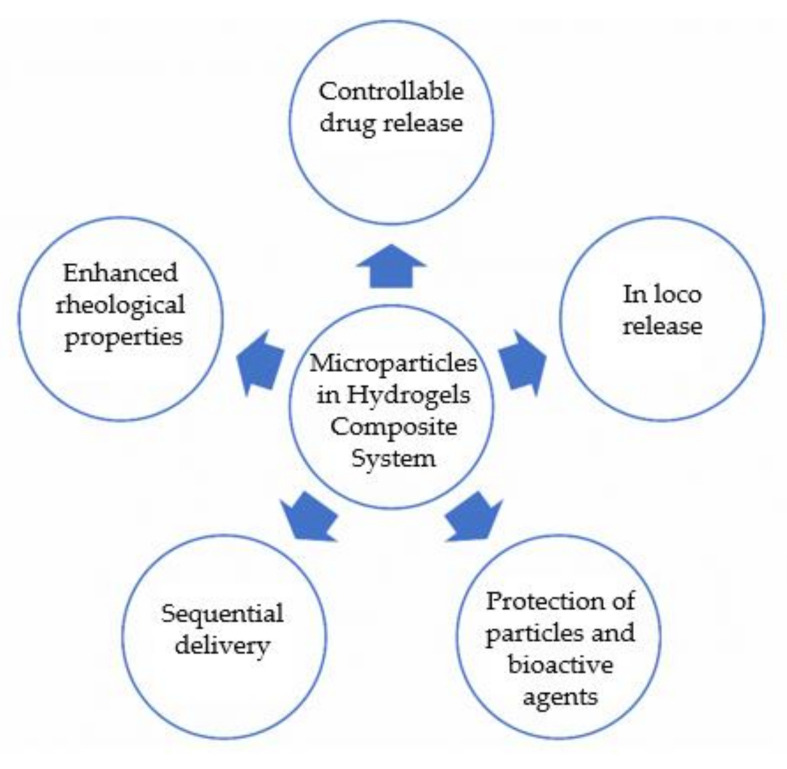
Advantages of microparticles in hydrogel systems for application as DDS.

This review will focus on in situ injectable composite DDS for biomedical applications based on a combination of microparticles and hydrogels. Herein will be described the reported advantages of this composite system (Figure 2) compared to microparticles and hydrogels alone, their design, and a compilation of recent studies that use this type of systems. This article will not focus on hydrogels and microparticles alone since several reviews analyse each component in more detail [8,12]. For the same reason, this article will not focus on the combination of hydrogels and nanoparticles; this combination can be found in some interesting review papers [13,14]. The focus of this review article is the combination of microparticles (at the micrometre scale) in hydrogels.

## 2. Microparticles in Hydrogel Systems

Designing an injectable microparticles in hydrogel system depends on several factors. This article focuses on an injectable system where a hydrogel loaded with microparticles is injected in situ. Due to the large diversity of microparticles and hydrogels, many possible combinations can be studied. In this case, the hydrogel will determine the system’s injectability, with the microparticles possibly changing certain mechanical characteristics of the hydrogel.

### 2.1. Gelation/Cross-Linking of the System

The system can suffer gelation in situ, meaning that the hydrogel will form the structure in vivo. Nonetheless, it is also possible for the sol-gel transition to occur before the injection. Regardless of the process, microparticles should be blended with the pre-gelation solutions in a homogenous manner so that the 3D gel structure has a homogenous particle distribution [14,15].

#### 2.1.1. In Situ Gelation

An example of in situ gelation is the use of thermosensitive hydrogels as matrices capable of sustaining the particles [11,16,17]. Before injection, at a temperature in which the hydrogel is in the sol state, the particles can be added, forming a suspension with the particles homogeneously distributed. After injection, the sol state will almost instantaneously change to the gel state, forming a structure with homogenously distributed particles in situ. 

Injecting the blended system with a cross-linking agent is also a possibility for in situ gelation. In this case, active ingredients that induce cross-linking of the precursor hydrogel mixture would also be injected. Of course, the mixture of the precursor reagents must be carried out immediately before the injection for the cross-linking to occur in vivo. Reaction time should also be considered because a quick gelation can influence its injectability [18].

Microparticles can also deliver the cross-linking agent to the hydrogel’s precursors. Using alginate microcapsules as calcium reservoirs, Hori et al. [19] developed an injectable in situ ionic cross-linking alginate system, knowing that alginate rapidly forms a 3D structure in the presence of divalent ions, especially Ca^2+^. Microcapsules loaded with calcium ions would be mixed with an alginate precursor solution and, when injected, the capsules would release the calcium ions, causing gelation of the alginate solution. This system released calcium ions within the first minutes, leading to a quick gelation. Another gradual hydrogel cross-linking formation is the release of calcium cations from CaCO_3_. A system of alginate-based hydrogels with hydroxyapatite and gelatine microspheres was developed by Yun et al. [20]. CaCO_3_ and glucono-d-lactone were used as gelation triggers. Hydrolysis of glucono-d-lactone would release protons that reacted with CaCO_3_, releasing calcium ions provoking alginate hydrogel formation.

#### 2.1.2. Gelation before Injection

It is also possible to cross-link hydrogels before injection. Hydrogels can be injected by having shear thinning behaviour, decreasing their viscosity with the applied shear rate, thus allowing the injection of already cross-linked hydrogels [21,22]. Having thixotropic behaviour is also advantageous because, with the lag time until the recovery of the original viscosity, the hydrogel will rearrange itself to best fit the implanted zone [23]. Self-healing hydrogels can repair structural damages and recover original functions and can also be used for this purpose [24].

### 2.2. Microparticle Mixture with the Hydrogel

**Figure 3:**
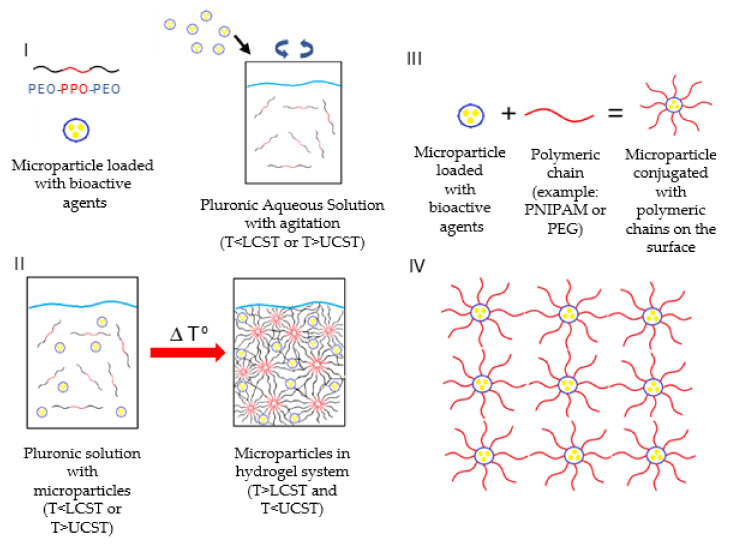
Thermoresponsive microparticles in Pluronic hydrogel system: (**I**) blending of Pluronic aqueous solution with microparticles, (**II**) microparticles in Pluronic hydrogel system gelation with particles evenly spread in the structure. Self-forming hydrogels with microparticles as cross-linking points: (**III**) the microparticles surfaces are conjugated with a polymeric chain (in red), (**IV**) the added chains will link to other chains forming a 3D structure with the microparticles serving as linking points.

Blending is the most common form to prepare these composite systems. Microparticles already loaded with drugs/bioactive agents are mixed with the hydrogel at the sol phase or with the hydrogel’s precursors [11]. The mixture should be prepared carefully to avoid any damage to the particles or the cargo. The particles must be evenly spread throughout the hydrogel matrix for a homogenous release. For example, Pluronic is a thermoresponsive polymer that presents a sol-gel transition at a certain temperature/composition binomial. Evenly distributing the particles in a Pluronic aqueous solution at lower temperatures (Figure 3I) followed by an increase in temperature will create a hydrogel with homogenously dispersed particles (Figure 3II) [11].

However, instead of having the microparticles separated from the hydrogel, the surface of the microparticles can be modified to form the matrix (Figure 3III) [25]. The microparticles will serve as crosslinking centres, avoiding blending steps and simplifying the systems (Figure 3IV). Zhao et al. [26] used a poly(L-lactic acid)-*b*-poly(ethylene glycol)-*b*-poly(N-iso-propyl acrylamide) copolymer (PLLA-PEG-PNIPAAm) to develop self-assembling nanofibrous gelling microspheres that could form a thermosensitive hydrogel for in situ injection. The PLLA branch of the polymer would form the microspheres, and PEG would provide hydrophilicity. At a physiological temperature, PNIPAAm would serve as physical cross-links between particles integrating the microspheres in the hydrogel matrix. In another study, Wang et al. [27] also developed copolymer microspheres with acrylamide and 2-hydroxyethyl methyl acrylate. Polymerizable double bonds were introduced to the surface of these microspheres. Then, these were dispersed in an acrylamide solution and with potassium persulfate to initiate the polymerization reaction and form a 3D structure. Compared with the original hydrogel, the system had higher compression strength and endured better strain tests.

### 2.3. Particle Release: Mesh Pore Size and Degradation of the Matrix

Hydrogels’ mesh pore size has a great widespan both in length and radius. If the hydrogel carries particles/bioactive agents with smaller sizes than the mesh size, diffusion will dominate the release mechanism. The smaller the particles/bioactive agents are compared to the mesh size, the quicker the particle/bioactive agent release will be. However, with increasing sizes, particles/bioactive agents will become more immobilized within the matrix. In this case, the release will be defined by changes in the mesh size/structure [12].

Degradation of the matrix, usually caused by hydrolysis or enzymatic activity, will break the bonds and expose the particles. Degradation can occur in bulk or only at the surface. However, since many of the hydrogels are permeable to water, bulk degradation is more common. Swelling can also be another form of release, where mesh sizes increase, letting drugs/particles out. This behaviour is caused by external conditions, such as pH or temperature, to which many hydrogels are sensitive. Another form of release is through mechanical deformations. Reacting to outside forces, the hydrogel’s structure will fall apart, causing the release of the particles/bioactive agents [12].

Moura et al. [28] compared the use of liposomes and chitosan microspheres for cisplatin transportation in a chitosan hydrogel system. In in vitro release studies, the liposome/hydrogel system released cisplatin very quickly, even when compared with plain hydrogels loaded with cisplatin. Drugs were added during the preparation of hydrogels, so it might be possible that they were permanently immobilized with crosslinking. The encapsulation of drugs within liposomes prevented drug interaction with the chitosan’s matrix, which might explain the faster release of the drug with liposomes. However, with chemically cross-linked hydrogels, the presence of chitosan microspheres significantly delayed the release and the initial burst compared with the free-form hydrogel.

The crosslinking density of the hydrogel and the addition of particles are of great importance. A higher cross-linking density will diminish swelling capacities, and therefore liquid penetration to the matrix. This causes a more difficult release pathway and retards it. Higher cross-linking densities also reduce loading capacities to the matrix. Karamzadeh [29] used polycaprolactone (PCL) microspheres/PEG-based hydrogels for the delivery of methadone. Two types of hydrogels were used to compare cross-linking agents. The one that gave a higher cross-linking density had a more delayed release. The introduction of PCL microspheres also significantly delayed the initial burst and the release profile. The system that used the hydrogel with the highest cross-linking density and microspheres was the one presenting slower release.

### 2.4. Surface Charge and Hydrophobicity of the Polymers

Polymers with opposite charges will create a physical bond due to electrostatic interactions. In a particle/hydrogel system, these interactions can strengthen the bonds between the two components of the system. França et al. [30] combined nanoporous silicon microparticles with a hydrogel of oxidized hyaluronic acid and adipic acid dihydrazide. Results showed that an electrostatic interaction occurred between the negatively charged Si-OH and Si-O-Si of the microparticles and the positively charged nitrogen bonds of the hydrogel, thus strengthening the microparticles in hydrogel mix. With microparticle presence, there was an improvement in mechanical properties and drug release kinetics in comparison with the pure hydrogel.

Another interaction that must be accounted for is the drug interaction with hydrophilic or hydrophobic polymers. The use of hydrophobic polymers must be evaluated when designing these systems. This will also depend on the desired cargo hydrophilicity or hydrophobicity. As stated above, the use of hydrophobic polymers allows the delivery of low water-soluble drugs. For example, using poly lactic-co-glycolic acid (PLGA) allows the regulation of the polymer’s hydrophobicity by changing the ratio between glycolic acid and lactic acid. An increase in the hydrophobicity of the system will cause a slower degradation rate, thus slowing the delivery rates of hydrophobic drugs. However, lower incorporation of drugs into the system will also occur [31].

### 2.5. Rheological Properties

**Figure 4:**
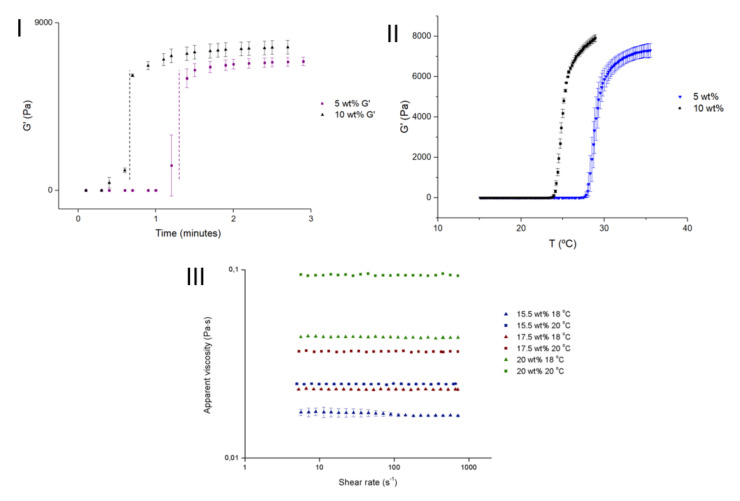
(**I**) Transient dynamic test with different particle concentrations; (**II**) temperature sweep in a dynamic test of a microparticles in hydrogel system with different particle concentrations; (**III**) flow curve of a microparticles in hydrogel system with different particle concentrations and at different temperatures. (Adapted with permission from [11]. Copyright (2021) MDPI).

Rheological properties are fundamental characteristics to understand in any type of injectable system, and the introduction of microparticles within a hydrogel may affect rheological properties. An example of this is the sol-gel transition of thermoresponsive hydrogels, which are commonly used as injectable systems. The presence of particles within a thermoresponsive hydrogel is reported to decrease the gelation time (Figure 4I). With hydrophilic particles, it is possible that the absorption of water from the hydrogel’s matrix to the particles leads to an increase in the hydrogel’s matrix concentration, decreasing its gelation time [11,23]. Additionally, an increase in the crosslinking density of the hydrogel caused by the presence of particles contributes to this phenomenon. Using a Pluronic F127 hydrogel (15.5 wt.%), the presence of alginate microspheres decreased the gelation temperature. Additionally, the increase in particle concentration from 5 wt.% to 10 wt.% further decreased the gelation temperature (Figure 4II). This may be due to a network formation with hydrogen bonds between carboxyl groups from alginate and ether groups from Pluronic [11]. The most common way of determining the gelation point is the crossover point between the storage and loss moduli in a temperature sweep test in Small Angle Oscillatory Shear (SAOS). Alighaie et al. [16] found that the addition of microspheres very slightly increased the temperature of modulus crossover in a microparticles in hydrogel system. However, using Fredrickson and Larson theory they found a decrease in the transition temperature with the presence of microparticles.

The presence of particles can also affect the gel-like structure by increasing the elastic behaviour of the material. With the introduction of gelatine microparticles within a hyaluronic acid hydrogel, Tsarik et al. [32] reported an increase in the moduli in a frequency sweep. They also studied the effect of injection on the gel structure. With a 16 G needle, both moduli of the hydrogel decreased, indicating a loss of elastic properties. Although a decrease was also observed in the microparticles in hydrogel system, the system still had higher moduli after injection than the hydrogel alone, thus preserving a gel-like structure. Delgado et al. [11] obtained an increase in both moduli with increasing PCL microcapsule concentration (5 wt.% and 10 wt.%) in a Pluronic hydrogel. The increase in the elastic behaviour of the composite system will depend on the concentration of microparticles used. Yan et al. [20] analysed different concentrations of gelatine microparticles in an alginate/hydroxyapatite gel. With increasing particle concentrations, the elastic modulus increased; however, at lower concentrations (2.5 wt.%), no increase in the modulus was reported compared to the hydrogel alone.

Apparent viscosity increases with the addition of particles due to an increase in structural integrity, leading to a more elastic material (Figure 4III) [10,11]. A more structured material will also affect the elastic modulus by increasing it.

As previously stated, the composite systems can suffer gelation before being injected. Therefore, the shear-thinning behaviour of the hydrogel is needed for the system to be injected. It is reported that the presence of particles does not affect the shear-thinning behaviour of the hydrogel [32].

### 2.6. Swelling

The swelling behaviour of the hydrogel system typically decreases with the introduction of microparticles. Depending on the interaction with the hydrogel’s chains and their concentration, the presence of microparticle can promote physical cross-linking of the structure, leading to reduced swelling. Additionally, due to the occupation of microparticles within the porous structure, less space will be available for water retention [23,33]. This means that microparticles can decrease the porosity of the hydrogel [34]. However, this decrease depends on the nature of the microparticles. It has been reported that these systems can also lead to an increase in swelling capabilities. The use of hydrophilic gelatine/heparin microspheres in a covalently cross-linked carboxymethyl chitosan/oxidized chondroitin sulphate hydrogel increased the swelling capabilities of the composite, likely due to the hydrophilic nature of the microparticles, that will also absorb some water [35]. Microparticles can also have no effect on swelling behaviour. Liu et al. [36] found no significant changes in swelling behaviour with PLGA microparticles introduction in a poly(ethylene glycol)-*co*-(L-lactic-acid) diacrylate/N-isopropyl acrylamide (PEG-PLLA-DA/NIPAAm) hydrogel, but this may be explained by the low concentrations of particles that were used in this study. As a consequence of the decrease in swelling, less liquid will penetrate the matrix, leading to a reduction in bulk degradation rate. Additionally, if the microspheres improve the cross-linking density, a more structured system will form, leading to higher degradation times.

## 3. Microparticles in Hydrogel Systems as DDS

### 3.1. Controllable Drug/Bioactive Agents Release Rate

DDS must maintain drug/bioactive agent concentration in situ within a certain range during the treatment. Very low concentrations will not be sufficient to maintain the desired therapeutic effects. However, higher concentration levels could originate undesirable cytotoxic side effects, causing more harm than good. In any delivery system, the ideal delivery profile should be a constant release rate, making it predictable. However, in most cases, this is not the case. Using only microparticles, a constant profile is difficult to achieve. A common feature in many types of microparticles is the “initial burst” effect. Basically, in a short period of time, large amounts of the loaded drug/bioactive agents are released, followed by lower release rates that are unable to maintain therapeutic concentrations. The addition of barriers can retard this effect, creating more diffusion pathways for the bioactive agents to bypass before being released in the in vivo environment [37]. Additionally, injecting drug/bioactive-loaded microparticles directly into the human body will expose them directly to the physiological environment, accelerating their degradation, which will promote the unloading of its cargo, accelerating the release. Embedding microparticles within a hydrogel can slow their biodegradation and retard the release.

**Figure 5:**
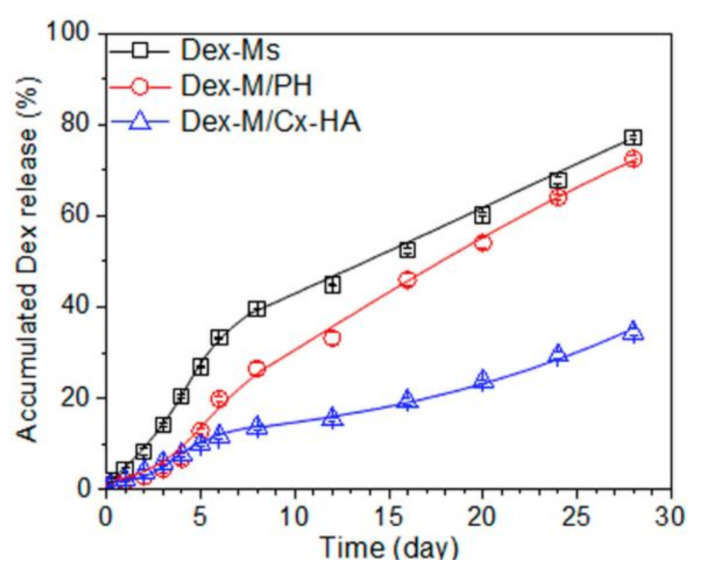
Accumulated in vitro release of DEX of microparticles and hydrogel alone and the composite system. (Adapted with permission from [38]. Copyright (2019) MDPI).

When compared to the release patterns of microparticles alone, the microparticles in hydrogel systems will have a more controlled release. Heo et al. [38] loaded dexamethasone (DEX) into PLGA microspheres and then combined them with Pluronic hydrogel and a hyaluronic acid (HA)-based hydrogel created via click reactions. The composite systems prolonged the delivery time and retarded the initial burst compared to the DEX loaded microspheres alone. In in vivo drug release tests, microspheres alone released 18% of DEX at day 1, 44% at day 7, and 83% at the end of 28 days, while the microspheres/Pluronic system released 3.2%, 32%, and 78% on days 1, 7, and 28, respectively. The initial burst release was delayed, with a significant decrease in the release profile on the first day. This may be due to the interaction of DEX with the lyophilic parts of Pluronic chains after the release from the microspheres. The following rapid degradation of Pluronic led to the rapid clearance of DEX, with higher cumulative releases with time. The microspheres/HA-based system released 4.7%, 14.4%, and 42% on days 1, 3, and 28. This system was capable to resist and release for long periods and retard the initial burst, maintaining therapeutic levels for longer periods (Figure 5).

The microparticles in hydrogel systems can also release at much slower rates when compared to simple hydrogels with homogenously distributed drugs. Our research group studied the combination of methylene blue-loaded alginate particles in a Pluronic hydrogel. The release of methylene blue from alginate microspheres embedded in a Pluronic hydrogel (15.5 wt.%) was 10 times slower when compared to methylene blue-loaded Pluronic hydrogel alone. Methylene blue was completely released from Pluronic hydrogel after 24 h. However, in the same period, only 38.2% was released from the composite system and complete release was only achieved after 240 h (10 days) [10,11].

Osswald and Kang-Mieler [39] studied the release profile of anti-vascular endothelial growth factor (VEGF) agents (ranibizumab or aflibercept) from PLGA microparticles from PNIPAAm based thermoresponsive hydrogel. They compared the release profiles and biodegradation in a phosphate-buffered saline solution at 37 °C of PLGA microparticles with the microparticle/hydrogel composite system. The microparticles alone degraded after 150 days. However, the microparticles in the hydrogel system lasted more than a month longer, at 196 days. Additionally, the composite system had a more prolonged release with a significantly more controlled burst release effect. With the delivery of ranibizumab, the initial burst effect released 50.3% from microparticles alone, compared to only 21% from the composite system. This difference was more pronounced with aflibercept, for which the microparticles had an 83.3% initial release, compared to a significantly lower 20.1% from the composite system.

### 3.2. In Loco Stability and Drug Release

**Figure 6:**
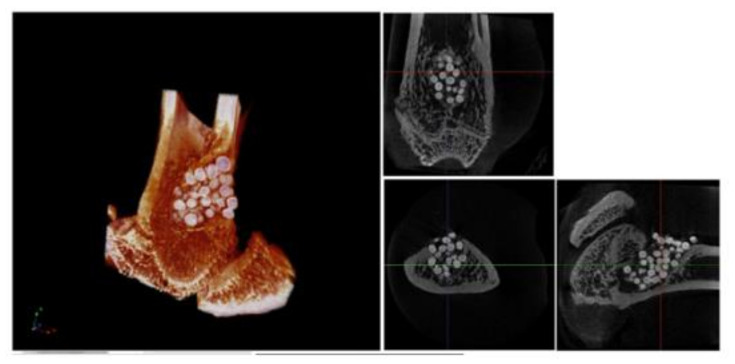
Micro-CT analysis of distal femur after 60 days of implantation. 3D reconstructed image and respective orthogonal slices of micro-CT acquisition of the femur with Sr-hybrid filled defect. (Adapted with permission from [40]. Copyright (2021) National Library of Medicine).

A controlled release pattern in situ will prevent the spread of drugs/bioactive agents to other parts of the body. For example, chemotherapeutic drugs are beneficial for the treatment of tumours, but also damage good tissues and are known to deteriorate the quality of life of patients. Ideally, the DDS in situ should only release the necessary quantities of bioactive cargo to the targeted area without spreading to neighbouring healthy tissues. Lourenço et al. [40] designed a system for local delivery of strontium, that orally administered can lead to cardiovascular-related problems, for bone tissue engineering. The researchers used a hyaluronic acid microparticles/alginate hydrogel system for in situ delivery (Figure 6). In vivo release studies suggested that strontium was only released into the defected site with no significant concentrations found in other organs.

### 3.3. Sequential Release and Co Delivery

**Figure 7:**
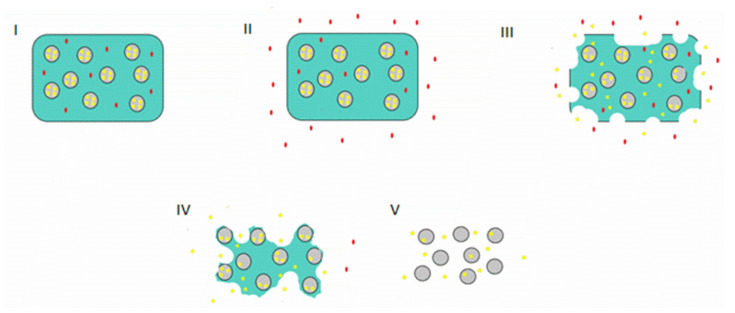
Scheme of a sequential delivery (**I**) microparticles in hydrogel loaded with two drugs (red in the hydrogel and yellow within the microparticles), (**II**) the red drug begins to be released at an early stage, (**III**) the hydrogel starts to degrade and the yellow drug diffuses trough the hydrogel, (**IV**) the red drug is completely released with the hydrogels degradation, (**V**) the microparticles stop being protected by the hydrogel and the yellow drug will be fully released with the complete degradation of the particles.

These systems can deliver more than one bioactive agent and in sequential order. Medical treatments, regardless of the area, are complex and have different phases. It is desirable to design a system that can deliver more than one drug/bioactive agent (Figure 7I), but also one that can release different drugs at different stages of the treatment, delivering the most suitable agent at each stage. In a microparticles in hydrogel system, it is possible to execute such sequential delivery. The hydrogel is the first one to be degraded. So, the cargo loaded in it will be the first one to be released and act (Figure 7II,III). As previously stated, the microparticles within the matrix will have their releases delayed. Thus, their cargo will be unloaded only after the first one (in the hydrogel) has started to be released and acted (Figure 7III,IV). Other particles can also be loaded with different release patterns (faster or slower) to add more delivery stages. Thus, it is possible to create a cascade treatment with progressive unloading of drugs that accompany the treatment stages [14,41,42,43,44].

Wound healing is a good example where sequential treatment can be beneficial. Four main stages define this healing process: haemostasis, inflammatory, proliferative, and maturation phases, in order. Ma et al. [41] developed a system comprised of a sodium alginate/bioglass hydrogel loaded with sodium alginate microparticles that, in turn, contained PLGA microparticles. The hydrogel would rapidly release ionic products from the bioglass that would control the inflammatory phase. In the following days, alginate microparticles loaded with a conditioned medium of RAW 246.7 cells would release their cargo to promote the formation of vascularized granulation tissue in the proliferation phase. Lastly, the PLGA microparticles would release pirfenidone to reduce fibrosis and prevent scar formation. In vivo studies in a mouse skin damage model were performed and it was observed that this system accelerated skin regeneration with less scar formation. 

**Figure 8:**
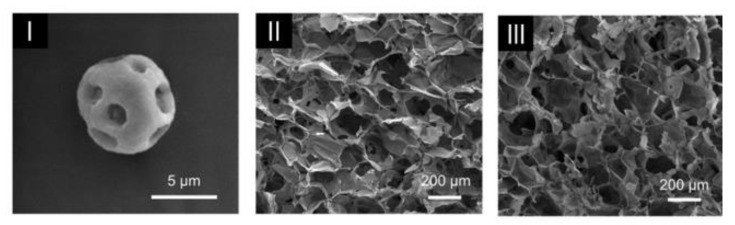
SEM images of (**I**) microparticle; (**II**) hydrogel; and (**III**) microparticles in hydrogel. (Adapted with permission from [42]. Copyright (2021) KeAi).

Myocardial infarction is a cardiovascular disease that causes damage and necrosis in myocardial cells. A sequential injectable silk fibroin microspheres in an alginate hydrogel system was designed by Wu et al. [42] for sequential co-delivery of VEGF and bone morphogenetic protein 9 (BMP9). The former promotes the proliferation of endothelial cells and angiogenesis, and the latter prevents myocardial fibrosis (Figure 8). In vitro release studies revealed that the loading of BMP9 to microspheres delayed its release. VEGF had a faster release than BMP9 due to its loading in the hydrogel. In vivo results showed that the composite was the system presenting the best performance in cardiac function improvement. Additionally, when compared with the free-form hydrogel loaded with only a single factor, the microsphere in hydrogel system with both factors improved cardiac function.

### 3.4. Encapsulation of Hydrophobic Drugs

Due to the hydrophilic nature of standard hydrogels, hydrophobic biological agents are not easily loaded within their structure. Therefore, hydrogels have been mostly restrained to the use of water-soluble drugs. There have been multiple strategies to incorporate hydrophobic drugs within the structure of the hydrogel. For example, in thermosensitive hydrogels composed of triblock polymers, the presence of a hydrophobic chain permits the formation of hydrophobic micelles. These can be used as reservoirs for drug transport. With these copolymers, hydrophobic drugs have a more prolonged release profile, whilst hydrophilic drugs have a more rapid one [45]. Another strategy is the incorporation of transport agents within the matrix, such as liposomes [46], niosomes [47], polymeric nanoparticles, and many others [48]. Thus, conventional hydrophilic hydrogels mixed with these particles can be used to deliver bioactive agents that they alone could not. Microparticles can also serve as hydrophobic drug carriers within hydrogels [18,49]. Hydrophobic polymers such as PLGA, polylactic acid (PLA) and PCL as carriers allow these drugs to be delivered in vivo. However, unlike hydrophilic drugs, these will be mainly released by particles erosion and not by diffusion [45].

Liu et al. [17] used a PCL–PEG–PCL copolymer to develop microspheres and thermosensitive hydrogels. It was found that dispersing the hydrophobic camptothecin in the sol state of the hydrogel promoted the formation of aggregates. So, they incorporated the drug within microspheres and these within hydrogels. Arunkumar et al. [50] studied the delivery of a highly hydrophobic drug, etoricoxib, to treat osteoarthritis. They compared the cumulative release of this drug from a chitosan hydrogel, PCL microparticles and a microparticles in hydrogel system of the two. Total release from the hydrogel happened within a week, whilst release from the microparticles and the composite system was prolonged for 30 days. Between these last two, the latter revealed to have a more controlled release profile of the hydrophobic drug than the microspheres. In in vivo clearance studies, the microparticle in hydrogel system also outperformed the microparticles and the hydrogel alone (the drug was still present for 6 weeks) and with low initial burst release. 

## 4. Fields of Application

The microparticles in hydrogel system has been used in different fields of biomedicine. The following chapter will focus on recent studies that used this type of composite system.

### 4.1. Cancer Treatment

Modern medicine has multiple cancer treatments available, including surgery, radiation therapy, immunotherapy, hormonal therapy and, most frequently, chemotherapy. Chemotherapy is the most-used therapy and consists of the application of cytotoxic drugs that can kill cancer cells, examples of which are doxorubicin (DOX), 5-fluorouracil (5FU), methotrexate (MTX) and paclitaxel. Unfortunately, direct application of drugs intravenously spreads these drugs throughout the body, leading to systemic toxicity and adverse side effects on the patient. Moreover, only a fraction of the administered drugs will reach the tumour site, and rapid clearance of the drugs will occur. Thus, to maintain therapeutic levels, higher doses and more administrations are required, exacerbating the problems. For example, chemotherapy is likely to attack hair follicles, leading to hair loss, and damaging blood-forming cells in the bone marrow, leading to anaemia and a weaker immune system, causing the patient to be more prone to other diseases and infections [51]. Furthermore, multi-drug resistance (MDR) is a significant problem in cancer management, with many of the initially responsive tumours developing resistance to multiple anticancer agents, giving rise to relapse [52]. In response to these issues, the composite system discussed here may provide a more controlled and enhanced therapeutic option. The microparticles in hydrogel system will be restrained to the tumorous area, allowing in situ drug delivery (Table 1). With this system, there is no need for high drug concentrations and repeated doses, reducing the side effects of chemotherapy. Moreover, this system can serve as a prevention system; after an operation, the cavity left by the tumour can be filled with this composite system to prevent tumour reoccurrence.

**Figure 9:**
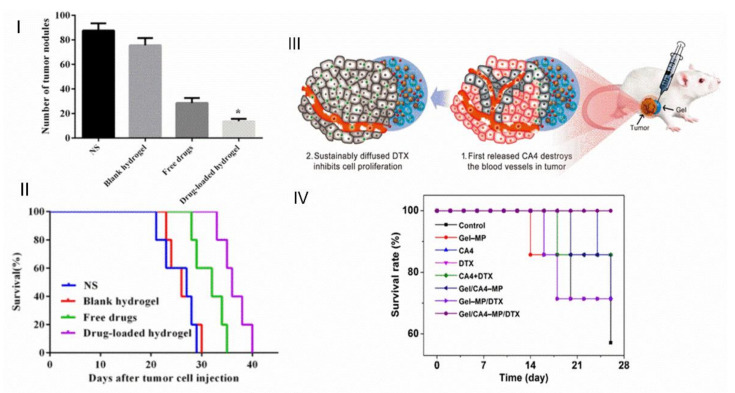
(**I**) Number of tumour nodules in each group; (**II**) survival in each group. (Adapted with permission from [54]. Copyright (2020) Elsevier). (**III**) Scheme of sequential codelivery in a tumour in vivo; (**IV**) survival rate of K7 osteosarcoma-grafted mice after treatment of PBS. (Adapted with permission from [14]. Copyright (2017) American Chemical Society).

A sequential release can be used in cancer treatment to deal with different stages of the treatment. Kim et al. [53] developed an injectable intratumoral sequential co-delivery system; it consists of PLGA microcapsules with DOX in their core and a thermosensitive hydrogel loaded with 5-FU. For hydrogels, they compared the behaviour of Pluronic with a poly(ethylene glycol)-b-poly(caprolactone-*co*-lactide) diblock copolymer. 5-FU would be released first from the hydrogel, followed by a later stage of DOX release. Comparing the two hydrogels, the diblock copolymer had slow biodegradation, whilst Pluronic quickly disappeared within 2 days of the administration, likely due to the disentanglement of its structures instead of biodegradation. The microcapsules in hydrogel systems performed better in vivo and in vitro than the isolated microcapsules and hydrogels (Figure 9I,II). In vivo drug biodistribution revealed that the drugs were confined in target tumours for 18 days, without spreading to normal cells. Concentrations below their toxic plasma concentrations would result in no toxicity damages. Luo et al. [54] developed a hyaluronic acid hydrogel with 5-FU and cisplatin and PCL-PEG-PCL microspheres loaded with paclitaxel. A sequential co-delivery system of chemotherapeutic drugs focusing on colorectal peritoneal carcinomatosis, an advanced stage of colorectal cancer, was possible with these two components. The hydrogel alone ultimately released the drugs within a week in vitro. However, the microspheres released only 40% of their content in the same period.

A thermoresponsive injectable system as a sequential co-delivery system (Figure 9III) was developed by Zheng et al. [14]; combretastatin A-4 (CA4) was loaded in a polypeptide hydrogel, and docetaxel (DTX) was loaded in PLGA microparticles. CA4 was expected to be released at an early stage to inhibit neovascularization (formation of new blood cells) and reduce the exchange of nutrients between the tumour and the surrounding tissues. Afterwards, DTX was expected to penetrate the new interstitial spaces and attack the surface cells of the tumour and inhibit cell proliferation. In vitro tests confirmed a quicker release of CA4 compared to DTX. It was also reported that the system did not suffer burst release of DTX, contrary to the PLGA microspheres. In vivo tests revealed that the total system degradation occurred after 42 days. The system was effective in inhibiting tumour cell proliferation. Histological and immunohistochemical analyses revealed that drug loading in these systems diminished their toxicity and prevented drug spreading outside the cancerous area. The survival rate of the composite system with co-delivery was also the highest compared to the other systems (Figure 9IV). The co-delivery of ibuprofen as an anti-inflammatory agent and DOX as a chemotherapeutic drug was also studied [55], resorting to an alginate hydrogel for ibuprofen early release and PCL microparticles for a later DOX release.

Zhu et al. [56] used vaterite microspheres embedded in silk nanofibers that formed a hydrogel for DOX delivery. The system had good injectability; the composite system resolved the poor water dispersibility of vaterite microparticles and the low drug release in silk nanofibers. In vitro tests of MCF-7 cells revealed that the composite system significantly decreased the viability of the cells. Ozeki et al. [57] also developed a system to treat brain tumours, using PLGA microspheres and a conjugated thermosensitive hydrogel of PEG and PNIPAAm. Camptothecin and vincristine were separately encapsulated in the PLGA microparticles, the former presenting a significant burst release causing systemic toxicity. The microparticles in hydrogel system with vincristine was more efficient, with increased survival rates at lower doses compared with the system with camptothecin.

### 4.2. Cardiovascular Diseases

Cardiovascular diseases are the most common causes of death globally, according to the World Health Organization [59]. Among them, myocardial infarction, more commonly known as a heart attack, occurs when areas of the heart are deprived of enough oxygen, generally due to the occlusion of a coronary artery, leading to a very significant cell loss. Developing new treatments that can promote angiogenesis and inhibit inflammatory responses to restore the structure and myocardium functions is critical. The composite system can serve as an excellent system for the treatment of these diseases (Table 2). 

M. Ciocci et al. [60] developed an injectable system as a scaffold for pre-cardiac cells to proliferate. A hydrogel made of silk fibroin was functionalized with poly(ethylene glycol) diacrylate to which albumin microparticles were embedded to form a microparticles in hydrogel system. In vitro 3D cell cultures, using cardiac mesenchymal stem cells revealed that this system was viable. The expression of protein characteristics at an early cardiac muscle cell differentiation were detected. The addition of chondroitin sulphate also improved cell proliferation within the composites.

**Figure 10:**
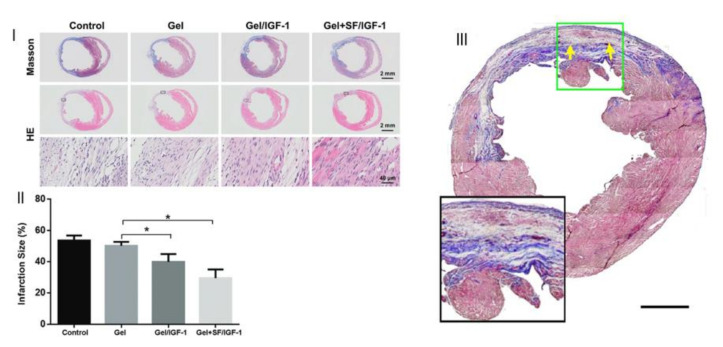
Fibrosis in the infarct area after induction of myocardial infarction. (**I**) Myocardial sections stained with Masson’s trichrome or H&E. (**II**) Statistical analysis of Masson’s trichrome staining 7 d post-MI (note: * *p* < 0.05). (Adapted with permission from [21]. Copyright (2020) Royal Society of Chemistry). (**III**) Representative Masson’s trichrome stained cardiac cross sections 16 weeks after injection of IGGF-1 loaded system (scale bar 500 μm). (Adapted with permission from [61]. Copyright (2013) American Chemical Society).

The addition of biological agents to systems like the one above can further stimulate cardiac muscle regeneration. Insulin-like growth factor 1 (IGF-1) is a hormone that can mediate several cellular processes within the heart and is correlated with the recovery of cardiac functions. Delivery of this agent can promote the recovery of cardiac functions after a heart attack. However, due to its short half-life, systemic injection is restricted, which led Feng et al. [21] to develop a composite system of an alginate hydrogel with silk fibroin microspheres for the sustainable delivery of IGF-1. The composite system had a more sustainable release when compared with the hydrogel and microspheres alone. After 28 days, a thicker wall and reduction in fibrosis occurred in the systems with IGF-1, the composite system presenting the lowest infarction size and higher improvement of cardiac function (Figure 10I,II). However, the presence of growth factors such as IGF-1 does not always have the desired outcomes. Nelson et al. [61] also used IGF-1 and basic fibroblast growth factors for myocardial infarction with hydrogels and microparticles. In in vivo conditions, high levels of these growth factors were present, but after two weeks, no added benefits were found in cardiac functions (Figure 10III).

Lyu et al. [62] coated PLGA microparticles with VE-cadherin fusion proteins and integrated them with human mesenchymal stem cells, thus creating functionalized aggregates for stem cell therapy. These 3D aggregates were expected to enhance cell proliferation and secrete bioactive agents to promote cardiac recovery and reconstruction. These aggregates were then encapsulated within a hyaluronic acid-based hydrogel via a Schiff base reaction between oxidized HA and hydrazide HA. The studied composite system prolonged the secretion of bioactive factors of the aggregates, with a reduction in collagen deposition and restored the left ventricular geometry.

### 4.3. Spinal Diseases

Spinal diseases affect our backbone, giving rise to many types of illnesses, from degenerative to traumatic, associated with this vital part of the human body. Microparticles in hydrogel systems have been studied as possible therapeutic tools to deal with those illnesses (Table 3). The composite system can be used as bioactive agent delivery structures and as scaffolds that can promote cell growth and regeneration of damaged tissue.

Minocycline hydrochloride (MH) and paclitaxel were co-delivered with a hemisection model of spinal cord injury in rats. Nazemi et al. [63] used an alginate hydrogel loaded with MH and PLGA microparticles with paclitaxel. MH is a small hydrophilic molecule that can be rapidly released from the hydrogel’s structure. The controlled release of this agent from the matrix was achieved using sodium alginate sulphate. The release of MH reduced the inflammatory activity within the damaged area, reducing its extension, while the release of paclitaxel promoted neuronal regeneration and a reduction in scar tissue. The system had a prolonged release up to 8 weeks, with a reduction in inflammation after 7 days. The regeneration of neurons and a reduction in scar tissue was also observed after 28 days. In another study [15], tannic acid was used to modify PLGA microparticles that encapsulated brain-derived neurotrophic factor (BDNF), and the microparticles were then embedded within a hydrogel of oxidized dextran and hyaluronic acid-hydrazide. The presence of tannic acid as an outer wall increased electrical conductivity and prolonged the release of BDNF due to the double barrier, thus forming a conductive injectable hydrogel, ideal for spinal regeneration. No burst effect was observed and the presence of tannic acid in the microspheres increased cell proliferation.

Intervertebral disc degradation is associated with chronic back pain. Tsaryk et al. [32] developed an injectable scaffold using collagen-low molecular weight hyaluronic acid with gelatine microspheres, aiming to develop a system that could be used in the recovery of the nucleus pulposus of intervertebral disks, preventing their degradation. The system supported the growth and chondrogenic differentiation of mesenchymal stem cells and the delivery of the growth factor TGF-b3 from the microspheres promoted the process.

### 4.4. Cartilage

Cartilage is a poorly vascularized tissue and has limited regenerative properties, meaning that cell migration and proliferation to damaged sites for regeneration are limited. Different strategies that focus on cartilage regeneration have been developed to cultivate and deliver cells and/or growth factors to the damaged areas that can then proliferate and differentiate to chondrocytes [65,66]. Microparticles in hydrogel systems can be an option to deliver cells, with Hydrogels offering a good proliferation structure and microparticles delivering the bioactive agents that can promote cell proliferation and chondrogenesis for cartilage regeneration (Table 4).

Bolandi et al. [67] used alginate sulphate beads within an alginate/PVA hydrogel for cartilage tissue engineering. Rabbit adipose-derived mesenchymal stem cells were encapsulated within the hydrogel with the microspheres carrying platelet-rich plasma (PRP). PRP is an excellent source of growth factors, and its sustained release within the vicinity of stem cells can promote cell proliferation and differentiation. PRP-loaded microspheres had higher cell proliferation than free PRP administration. In in vitro gene expression tests, the composite system had a much higher gene expression of Collagen II, Aggrecan, and SOX9 than the control and hydrogel with free PRP. This great contrast is attributed to the sustainable release of PRP from the microbeads and an increase in the half-life of growth factors. This proposed system eventually led to the differentiation of the delivered stem cells to chondrogenesis.

**Figure 11:**
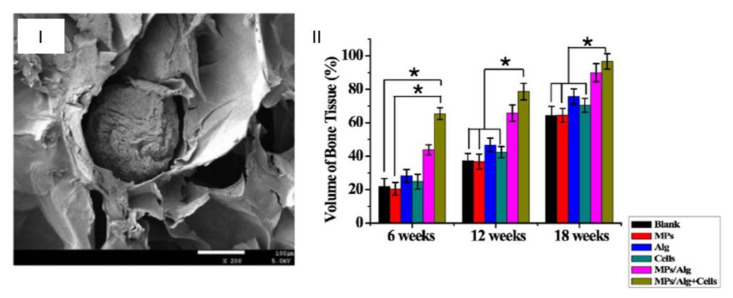
(**I**) SEM image of a cross section of porous microparticles within an alginate hydrogel (scale at 100 μm); (**II**) volume of newly formed bone tissue in vivo in different groups 6, 12, and 18 weeks after operation (* *p* < 0.05). (Adapted with permission from [70]. Copyright (2021) American Chemical Society).

Asgari et al. [68] introduced kartogenin, a small molecule that can induce chondrocyte differentiation of mesenchymal stem cells within PLGA microparticles. These were then incorporated into MSC aggregates to promote stem cell differentiation. An injectable gelatine methacryloyl hydrogel was used and loaded with curcumin to promote an anti-inflammatory response and diminish hypertrophy to provide a scaffold. After 12 weeks, the system loaded with curcumin presented the best regeneration of the cartilage. Chen et al. [69] used PLGA particles with stromal cell-derived factor-1 within a chitosan–gelatine composite hydrogel. The microparticles in hydrogel system had longer release cycles compared to the hydrogel alone. Promotion of the repair of osteochondral defects was achieved by the incorporation of this agent in the composite system. Porous microparticles have also been used to promote cartilage regeneration; Liao et al. [70] loaded PCL-PEG-PCL porous particles with calcium gluconate for in situ cross-linking of an alginate gel (Figure 11I). The system served as an injectable scaffold for cartilage regeneration within a cartilage defect in vivo. An almost-complete repair occurred after 18 weeks, with an optimal regeneration of cartilage tissue (Figure 11II).

### 4.5. Bone

Bone tissue engineering aims to promote natural bone regeneration. The delivery of bioactive agents that can promote bone formation is an extremely important research area. Microparticles in hydrogels systems have been used as a DDS of these agents for bone therapy, and herein some recent works are presented (Table 5).

**Figure 12:**
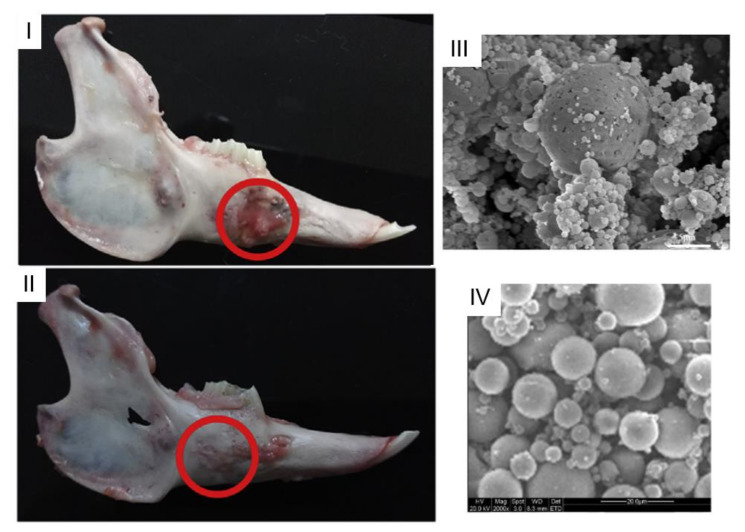
Observation of rabbit mandibles samples 12 weeks after operation (**I**) control group, (**II**) BMP-2/VEGF group, (**III**) SEM images of drug-loaded PLGA particles (scale 5 μm). (Adapted with permission from [74]. Copyright (2020) Elsevier); (**IV**) PDGF-BB encapsulated alginate microparticles. (Adapted with permission from [76]. Copyright (2019) MDPI).

VEGF promotes neovascularization and is very important for the growth of new capillary vessels. Bone morphogenetic protein-2 (BMP2) with osteogenic properties is used to promote osteoblast differentiation. This growth factor is widely used in bone tissue engineering. The co-delivery of VEGF and BMP2 for synergetic effects has been the topic of some research works [71,72]. In a study [73], PLGA microcarriers, prepared using microfluidics and loaded with VEGF, had their surface modified with poly(3,4-dihy-droxyphenethylamine) (PDA) for BMP2 conjugation. The microcarriers were cultured with mesenchymal stem cells (MSC), and then this mixture was embedded within an alginate-RGD hydrogel for injection. PDA prolonged bioavailability and enhanced the attachment and proliferation of MSC. This system had the potential of tunnelling cell formation, proliferation, and differentiation. The co-delivery of VEGF and BMP2 had better performance than the delivery of only one growth factor (Figure 12I–III). Wang et al. [74] loaded hydroxyapatite/PLGA microspheres with VEGF and BMP2. These carriers were embedded in an injectable chitosan scaffold. Adipose-derived stem cells were also embedded within the hydrogel. In vivo tests were performed on mandibular bone defects in rabbits. Nearly all scaffolds degraded within 8 weeks after injection. In systems with BMP2 and BMP2/VEGF, the defects completely healed within 12 weeks with the scaffold integrated into the tissue. Comparing these two systems, the co-delivery of VEGF and BMP2 showed the best performance. Liao et al. [75] also developed a composite system to deliver adipose stem cells with platelet-rich plasma for osteoinductive properties. An injectable hydrogel of hyaluronic acid and chitosan was embedded with the stem cells and biphasic calcium phosphate ceramic microparticles. The synergistic action of biphasic calcium phosphate and the platelet rich plasma proved to promote osteogenesis.

Sequential delivery of BMP2 and platelet-derived growth factor-BB (PDGF-BB) was studied by Min et al. [76]. PDGF-BB was aimed to be released first to recruit precursor cells and stimulate angiogenesis. Then, BMP2 would be released to promote osteogenesis. A chitosan/glycerophosphate thermosensitive injectable hydrogel was used as a matrix. PDGF-BB was loaded in alginate microspheres and BMP2 loaded in core–shell microparticles of chitosan-PLA with an alginate layer serving as a double barrier for later release (Figure 12IV). The sequential release was achieved with tuneable release rates regulated by the amount of loaded bioactive agents and microparticles. 

**Figure 13:**
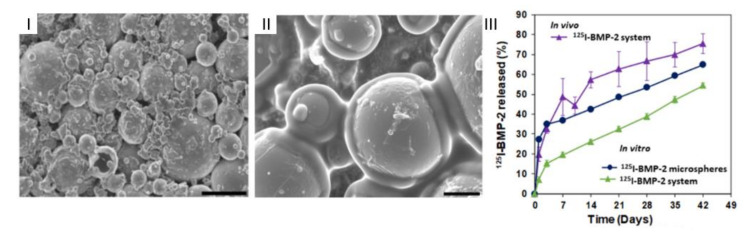
Microparticles embedded in a hydrogel: (**I**) composite system freshly prepared (scale 100 μm); (**II**) composite system after 4 weeks in water (37 °C, 25 rpm); (**III**) release tests of BMP-2: in vitro: from microparticles (blue) and the composite system (green), in vivo: composite system (purple). (Adapted with permission from [77]. Copyright (2021) MDPI).

Osteoporosis is a disease that deteriorates the bones, making them fragile, and a significant deterioration in the quality of life occurs. Women are much more impacted by this disease, especially when they reach menopause. After menopause, oestrogen deficiency leads to a decrease in bone restoration. To combat this oestrogen deficiency and promote bone regeneration, García et al. [77] developed a complex injectable “sandwich” system where BMP2 and 17β-oestradiol were incorporated. PLGA and PLA microspheres were used, along with a chitosan/collagen hydrogel and hydroxyapatite nanoparticles (Figure 13I,II). The composite system had a more delayed release (Figure 13III). The interaction of hydroxyapatite and BMP2 delayed the initial burst effect from microspheres. The obtained results suggested that only BMP2 influenced bone regeneration. Segredo-Morales et al. [78] combined BMP2, 17β-oestradiol and platelet-rich growth factors to treat osteoporotic bone defects. Using PLGA and PLA-S microspheres embedded in a poloxamine/alginate thermoresponsive hydrogel, a synergetic injectable system was produced. The addition of alginate increased the viscosity and reduced the gelation temperature of the poloaxamine.

Gong et al. [79] used an injectable hydrogel consisting of fibrinogen and thrombin, also named fibrin glue. This is used as a bio-adhesive in surgery and has potential in bone regeneration. CaCO_3_ microspheres functionalized with casein and heparin were embedded in this hydrogel with BMP2. A significant increase in tensile strength was observed with the sustained release of BMP2. In a rabbit tibia bone defect model bone, defects were nearly cured after 8 weeks with the composite system. 

### 4.6. Skin

As previously stated, wound healing is divided into four stages. After the trauma happens, the first phase is the haemostasis stage. This consists of blood vessel constriction and the promotion of platelet action to control bleeding. The inflammatory phase occurs after trauma and aims to clean the wound and eliminate foreign agents (bacteria, viruses, etc.). Thirdly, new tissue begins to be built with granulation tissue and angiogenesis at the proliferative stage. Lastly, the maturation stage evolves wound contraction through the action of myofibroblasts and reinforcement of the structure. Collagen type III will be replaced with collagen type I, the main component of the dermis. The composite systems can be injected into the damaged areas to accelerate the occurrences at each stage. Additionally, the hydrogel would adapt to the damaged area, providing a scaffold for more rapid wound closure [82] (Table 6).

**Figure 14:**
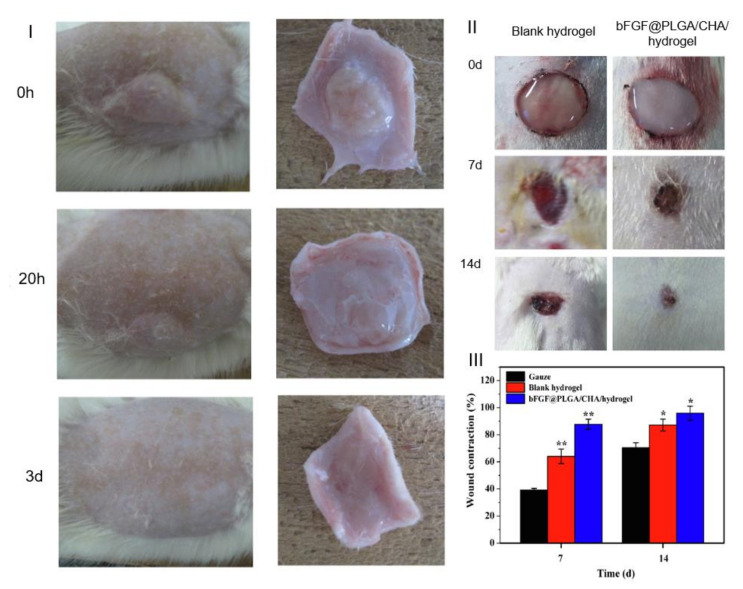
(**I**) Gross observation of in vivo degradation of the implanted hydrogels after subcutaneous injection (left) and the tissue surrounding the hydrogels extracted (right) from the rats, (**II**) gross observation of wound contraction of blank hydrogel (left) and loaded composite (left), (**III**) percentage of wound contraction. *p < 0.05, **p < 0.01 compared with the gauze group. (Adapted with permission from [83]. Copyright (2019) Elsevier).

As stated above, the composite system can deliver more than one bioactive agent with different agents for the four stages [41]. Chen et al. [83] developed a self-healing hydrogel for sequential delivery to prevent infections and promote wound healing. Chlorhexidine acetate (ChlHt), a known antibacterial agent, and BFGF were loaded into a composite system. ChlHt was dispersed in a hydrogel and BFGF inside PLGA microparticles. ChlHt was rapidly released from the system providing antibacterial properties to the damaged area. Then, BFGF was released, promoting tissue formation and accelerating regeneration (Figure 14I–III). Xu et al. [84] developed a chitosan thermoresponsive hydrogel with PLGA microspheres with platelet-derived growth factor receptors. The composite system had a better performance than the delivery of PDGF alone. The composite system promoted granulation formation and collagen deposition. In vivo tests revealed a more accelerated wound closure.

These systems have also been used for soft tissue augmentation for aesthetic purposes such as skincare and skin rejuvenation [85]. Ding et al. [86] loaded fibroblasts into PDLLA porous microparticles. The porous structure would allow attachment and promote the proliferation of fibroblasts. These carriers were then embedded in a collagen hydrogel, an ideal material for tissue regeneration. The hydrogel also provided a structure and nutrition for cell proliferation. The composite system had much higher cell viability than the microspheres alone, thus promoting skin regeneration.

### 4.7. Other

Microparticles in hydrogel systems have also been applied in many other different fields (Table 7). Osswald et al. [87] evaluated the delivery of anti-VEGF with PLGA microparticles in a PNIPAAm hydrogel for ophthalmic purposes. The use of this system revealed a significant reduction in ocular lesions. Mohammadi et al. [31] developed a sequential release system for post-operative ophthalmic uses; different triblock hydrogels were compared and loaded with PLGA microparticles, and three drugs were loaded. An antibiotic was loaded to the hydrogel for rapid release; to the PLGA microparticles, a steroid and an ocular hypotensive were loaded for a more prolonged delivery. The use of more hydrophobic polymers delayed degradation and release profiles but diminished the loading of the drugs.

Diabetes is a prevalent disease, and the need for constant insulin administration is a problem. Zhao et al. [22] developed a glucose-regulated composite system for insulin delivery using PLGA-based microparticles and hyaluronic acid-based hydrogel. The system was able to release insulin in response to high concentrations of glucose. With an in vivo model using diabetic mice, glucose levels remained quasi-stable for about two weeks.

For periodontitis, Wang et al. [88] used PLGA microparticles and a thermoreversible hydrogel of poly(isocyano peptide) to release doxycycline and lipoxin. PLGA microparticles were divided into acid-terminated and ester-capped. Acid-terminated particles degraded and released their content faster than the ester-capped particles; the latter had a triphasic release. Their combination with the hydrogel revealed that the systems that used more ester capped particles had a slower release.

A gelatine hydrogel with PLGA microparticles loaded with simvastatin has been used to prevent possible pathologies associated with tooth extraction [89]. The system had a more controlled release profile compared with the microspheres and hydrogel alone. Teeth repair and regeneration was observed after 5 weeks of implantation in rats. 

### 4.8. Tissue Engineering

So far, the article has focused more on the benefits of these systems in drug/bioactive agent release scenarios. However, it is important to notice that these systems can also serve as scaffolds for cell proliferation for tissue regeneration. Many hydrogels mimic the extracellular matrix of different tissues and are biocompatible. Hydrogels can serve as temporary 3D supportive structures for the formation of tissue. Thanks to their high-water uptake capacity and porous structure, cells can use them as scaffolds for their proliferation and eventual tissue formation. The use of hydrogels in tissue engineering as scaffolds has been extensively studied and multiple types of hydrogels can be used [90]. In a composite system, microparticles can serve as carriers of growth factors and regulators to promote the proliferation and differentiation of the cells [41].

Mesenchymal stem cells (MSC) are multipotent stem cells that can differentiate into cells from the cartilage, bone, and fat. These can be loaded into a hydrogel/microsphere system carrying also bioactive agents with specific growth factors that can help proliferation and differentiation. MSC can be mixed with microparticles and then injected within a hydrogel to a damaged area within the bone or cartilage. Microparticles can be loaded with biological agents that can promote MSC differentiation [67,73]. The structural aid of the hydrogel with the sustainable release of biological agents will promote MSC proliferation and differentiation, thus accelerating tissue regeneration. Naghizadeh et al. [66] encapsulated adipose derived stem cells (a subset of MSC) within an oxidized alginate and carboxymethyl chitosan hydrogel with chitosan microparticles. Chitosan was conjugated with melatonin to increase the permeability of the drug, and the microparticles also carried methylprednisolone for inflammation regulation. The system with the best cartilage regeneration was the one with microparticles. The composite system had a very significant expression of marker genes and a great amount of glycosaminoglycan detected when compared to the hydrogel alone.

## 5. Conclusions

The microparticles in hydrogel system is an ideal DDS that allows a more controlled release of bioactive agents and can be used in many different fields in biomedicine. This combination of microparticles with hydrogels might seem a relatively straightforward process; however, it creates a multipurpose system. It will be more durable when compared with microparticles or hydrogels alone, permitting more prolonged releases. Thus, it is important to determine the complete biodegradability of the system and its components. Additionally, different release patterns of bioactive agents are possible with this combination, allowing more complex treatments. The addition of microparticles will change the hydrogel matrix’s properties, such as the decrease in the gelation temperature in thermoresponsive hydrogels. Of course, the stability of the system is an important goal to achieve, and it is important to optimize and adjust the system to the desired treatment. In sum, the injectable microparticles in hydrogel system is an ideal DDS that can be developed to serve different purposes within the human body.

## Figures and Tables

**Table 1:** Microparticles in hydrogel system used for cancer treatments.

Microparticles	Hydrogels	Bioactive Cargo	Observations	Ref.
PLGA	PLAF-b- PEG-b- PLA	Combretastatin A-4 and docetaxel	Sequential release of drugs. The synergistic system suppressed the spread of an osteosarcoma in in vivo tests.	[14]
PCL–PEG–PCL	PCL–PEG–PCL	Camptothecine	The composite system had a strong anti-tumour effect. The system suppressed growth and metastasis in in vivo tests.	[17]
Double walled microparticles with PLGA and PDLLA	Aldehyde functionalized sodium alginate	Cisplatin and paclitaxel	Beyond the use for chemotherapeutics the hydrogel of the system also served as an antibacterial agent, due to the presence of polyethyleneimine within the hydrogel.	[49]
PLGA	Pluronic and copolymer of PEG-PCL-PLLA	DOX and 5 FU	Microparticles carried DOX and the hydrogel carried 5 FU. Pluronic rapidly cleared whilst the copolymer endured. The spread of the drug to healthy tissues was not observed in vivo.	[52]
PCL–PEG–PCL	Hyaluronic acid	5 FU, cisplatin and paclitaxel	Hydrogel loaded with 5Fu and cisplatin and microspheres with paclitaxel. Both evaluated separately. Good in vivo performance.	[54]
PCL	Alginate	DOX and ibuprofen	Early release of ibuprofen as an anti-inflammatory agent followed by DOX release for cytotoxic effect.	[55]
Vaterite microspheres	Silk nanofibers	DOX	Several combinations made. The system significantly decreased viability of MCF-7 cells in in vivo tests.	[56]
PLGA	PEG and PNIPAAm conjugates	Camptothecin and vincristine	Drugs loaded only on the microparticles. Vincristine had a better performance than camptothecin, with longer rates of survival.	[57]
Gelatine	Alginate and chitosan	5 FU and iron oxide nanoparticles	Under an external magnetic field, the system released higher quantities of 5 FU for higher times but had no effect in the initial release.	[58]

**Table 2:** Microparticles in hydrogel systems for cardiovascular diseases treatment.

Microparticles	Hydrogels	Bioactive Cargo	Observations	Ref.
Silk fibroin	Alginate	IGF-1	After 28 days microspheres in hydrogel system with IGF-1 had the higher enhancement in cardiac function.	[21]
PLLA-PEG-PNIPAAm	PLLA-PEG-PNIPAAm	Stem cells	Copolymer assembled into microspheres. PNIPAAm branch served has physical links that formed a hydrogel in the presence of water.	[26]
Silk fibroin	Alginate	VEGF and BMP9	Sequential release of two bioactive agents. VEGF was loaded to the hydrogel (for rapid release) and BMP9 to the microparticles (for prolonged release).	[42]
Albumin	Silk fibroin and PEGDA	Stem cells	In vitro 3D cell culture revealed proteins characteristic of early cardiac muscle cell differentiation.	[60]
PLGA	Poly (NIPAAm-co-HEMA-co-MAPLA) copolymer	IGF-1 and bFGF	In vivo presence of growth factors was high but no improvements to cardiac functions were noted.	[61]
Protein coated PLGA	Based-hyaluronic acid	Stem cells	Formation of functionalized mesenchymal stem cell aggregates for the delivery of bioactive factors with the protection of hyaluronic acid hydrogel.	[62]

**Table 3:** Microparticles in hydrogel systems for spinal cord diseases treatment.

Microparticles	Hydrogels	Bioactive Cargo	Observations	Ref.
PLGA with tannic acid	Oxidized dextran and hyaluronic acid-hydrazide	BDNF	Tannic acid increased electrical conductivity and mechanical properties.	[15]
Gelatine	Collagen-low molecular weight hyaluronic acid	TGF-b3	The hydrogels supported growth and differentiation of MSC. Microparticles delivered TGF-b3 that promoted cell differentiation.	[32]
Gelatine	Hyaluronic acid and N-isopropylacrylamide	Epigallocatechin 3-gallate	Reduction of inflammatory response with efficient drug encapsulation with electrospraying.	[44]
PLGA	Alginate	Minocycline hydrochloride and paclitaxel	Co-delivery of drugs. MH regulated the inflammatory process and PTX promoted tissue regeneration and decreased scar tissue.	[63]
PLGA	Hyaluronic acid and methylcellulose	Brain-derived neurotrophic factor (BDNF)	The composite system had a more controlled release of BDNF compared to the hydrogel alone.	[64]

**Table 4:** Microparticles in hydrogel systems for cartilage defects treatment.

Microparticles	Hydrogels	Bioactive Cargo	Observations	Ref.
Chitosan	Oxidized alginate and carboxymethyl chitosan	Melatonin and methylprednisolone	Melatonin conjugated with chitosan. Tripolyphosphate was used to develop microspheres loaded with methylprednisolone. In vivo tests compared the effect of hydrogel alone and microparticles in hydrogel system. The latter had the best cartilage regeneration.	[66]
Alginate sulphate	Alginate and PVA	PRP and Rabbit adipose-derived mesenchymal stem cells	Sustained release of PRP to the hydrogel carrying stem cells had a significant gene expression compared with the hydrogel with free PRP.	[67]
PLGA	Gelatine methacryloyl	Kartogenin and curcumin	Microparticles loaded with kartogenin were incorporated within MSC aggregates to promote chondrocyte differentiation and the hydrogel served has a scaffold with curcumin to moderate inflammatory response.	[68]
PLGA	Chitosan and gelatine	Stromal cell-derived factor-1	Injectable hydrogel for recovery of osteochondral tissue regeneration. The microparticles in hydrogel system had a prolonged release profile and promoted the cartilage regeneration.	[69]
PCL-PEG-PCL	Alginate and PVA	Calcium gluconate	Calcium gluconate served as an in situ cross-linking solution for the alginate hydrogel.	[70]

**Table 5:** Microparticles in hydrogel system for bone diseases treatment.

Microparticles	Hydrogels	Bioactive Cargo	Observations	Ref.
Hydroxyapatite	Alginate	Strontium	Strontium presence increased the bone formation. Only local delivery occurred.	[40]
Bioglass	PVA	BMP2 and VEGF	Increase in mechanical properties. In vivo tests revealed that the microparticles in hydrogel system is more efficient than bulk hydrogel to treat bone defects.	[71]
Chitosan	Methyl cellulose and alginate	BMP2 and VEGF	VEGF was loaded to the hydrogel and BMP within the particles. VEGF had a faster release when compared to BMP2.	[72]
PLGA	Alginate	BMP2 and VEGF	Injectable composite system for sequential delivery of bioactive factors for vascularization and bone regeneration. In vivo, the system induced vascularization and ectopic bone formation.	[73]
PLGA and hydroxyapatite	Chitosan	BMP2 and VEGF	After 12 weeks, the delivery of BMP2 and the co delivery of both agents using the composite system promoted a complete healing of the bone defect.	[74]
Biphasic calcium phosphate	Hyaluronic acid and chitosan	Adipose-derived stem cells and platelet rich plasma	Development of a thermoresponsive microparticles in hydrogel system. Improvement of cell proliferation and mineralization of extracellular matrix.	[75]
Alginate and chitosan/PLA/alginate	Chitosan and glycerophosphate	BMP2 and platelet-derived growth factor-BB	Sequential release with encapsulation in different microparticles. Release patterns regulated by the number of microparticles embedded in the hydrogel and the initial drug load in microparticles.	[76]
PLGA and PLA	Chitosan and collagen	BMP2 and 17β-estradiol	Presence of hydroxyapatite diminished burst effect on PLGA microspheres with BMP2. Results suggested that 17β-estradiol had no effect on bone regeneration.	[77]
PLGA and PLA-S	Poloxamine (T-1307) and alginate	BMP2, platelet rich growth factors and 17β-oestradiol	Alginate increased viscosity and diminished the gelation temperature of poloxamine. Microparticles increased viscosity. Drug release had an early fast release followed by a prolonged slow released to up 6 weeks.	[78]
CaCO3	Fibrin-glue	BMP2	Within 8 weeks in vivo in tibia bone defects of rabbits, the composite system had nearly healed the defects.	[79]
Gelatine	Synthetical polymers	Mesenchymal stem cells	Gelatine microparticles served as porogens for the hydrogel to promote cell attachment and proliferation.	[80]
Calcium carbonate microparticles and hydroxyapatite nanoparticles	Chitosan and alginate	Tetracycline hydrochloride	Hydroxyapatite and microparticles ratio affected mechanical properties. Sustained release and antibacterial properties were observed.	[81]

**Table 6:** Microparticles in hydrogel system for skin treatment.

Microparticles	Hydrogels	Bioactive Cargo	Observations	Ref.
PLGA and alginate	Alginate and bioglass	Condition medium of RAW 246.7 cells and pirfenidone	Sequential release that accompanies the different stages of wound healing, with specific agents for each one.	[41]
Alginate	Oxidized alginate and N-succinyl chitosan	Bull Serum Albumin	Higher compressive modulus and slower release profiles compared with the hydrogel and microparticles alone.	[82]
PLGA	Aminated gelatine, adipic acid dihydrazyde and oxidized dextran	Chlorhexidine acetate and basic fibroblast growth factor (bFGF)	Self-healing hydrogel with antibacterial properties and sequential delivery. Chlorhexidine acetate would serve as an antibacterial agent for an early release, followed by a latter release of bFGF for cell proliferation.	[83]
PLGA	Chitosan	Platelet-derived growth factor receptor	Promotion of granulation formation and collagen deposition with the microparticles in hydrogel system.	[84]
Hydroxyapatite	Hyaluronic acid	None	Application for rejuvenation of aged skin. Viscosity and storage modulus increased with hydroxyapatite microparticles presence.	[85]
PDLLA	Collagen	Fibroblasts	Porous PDLLA microspheres served as scaffolds for cell adhesion	[86]

**Table 7:** Microparticles in hydrogel system application in diverse treatments.

Treatment Objective	Microparticles	Hydrogels	Bioactive Cargo	Observations	Ref.
Diabetes	PLGA-based	Dopamine-conjugated hyaluronic acid	Insulin	Glucose-regulated insulin delivery system. With a single administration, stabilized glucose’s level for 2 weeks in vivo.	[22]
Post-operative ocular system	PLA	Triblock polymers	Fluoroquinolone moxifloxacin, dexamethasone, and beta-blocker levo-bunolol	Different triblock polymers were studied. Hydrophobicity used to regulate the delivery profile and to delay the initial burst.	[31]
Macular degeneration	PLGA	PNIPAAm	Anti-VEGF	Prolonged drug delivery. In vitro release prolonged for about 200 days.	[39]
Macular degeneration	PLGA	PNIPAAm	Anti-VEGF	The microparticles in hydrogel system decreased the ocular lesion areas in vivo.	[87]
Periodontitis	PLGA	Polyisocyanopeptide	Doxycycline and lipoxin	Ester capped PLGA microparticles had a longer release profile than the acid terminated ones	[88]
Periodontitis	PLGA	Gelatine	Simvastatin	Controlled release of simvastatin for tooth extraction. The microparticles in hydrogel system promoted bone formation after 5 weeks.	[89]

## Data Availability

Data are contained within the article.
